# The KasA inhibitor JSF-3285 improves the sterilizing activity of bedaquiline-pretomanid-containing regimens in a mouse model of tuberculosis

**DOI:** 10.1128/aac.00130-25

**Published:** 2025-04-23

**Authors:** Heena Soni, Sandeep Tyagi, Kishor Mane, Anil M. Shelke, Pradeep Kumar, Firat Kaya, David Alland, Matthew Zimmerman, Joel S. Freundlich, Eric L. Nuermberger

**Affiliations:** 1 Center for Tuberculosis Research, Department of Medicine, Johns Hopkins University 1466, Baltimore, Maryland, USA; 2 Department of Pharmacology, Physiology, and Neuroscience, Rutgers University–New Jersey Medical School 67206 , Newark, New Jersey, USA; 3 Division of Infectious Disease, Department of Medicine and the Ruy V. Lourenco Center for the Study of Emerging and Re-emerging Pathogens, Rutgers University–New Jersey Medical School, Newark, New Jersey, USA; 4 Public Health Research Institute, Rutgers University–New Jersey Medical School, Newark, New Jersey, USA; 5 Center for Discovery & Innovation, Hackensack Meridian Health, Nutley, New Jersey, USA; St George's, University of London, London, United Kingdom

**Keywords:** *Mycobacterium tuberculosis*, antitubercular, KasA, mouse relapse model, JSF-3285

## Abstract

JSF-3285 is a promising preclinical candidate for tuberculosis that potently targets the *Mycobacterium tuberculosis* β-ketoacyl-ACP synthase KasA. In mouse models of acute, sub-acute, chronic, and relapse infection, JSF-3285 offers substantial activity in combination with bedaquiline and pretomanid, which could be applicable for both drug-sensitive and drug-resistant infections.

## INTRODUCTION

Tuberculosis (TB) is a global health threat with an estimated 10.8 million new cases occurring in 2023 (1). An estimated 400,000 of these new cases involved rifampicin-resistant or multidrug-resistant (RR/MDR) *Mycobacterium tuberculosis*. The current standard of care for drug-susceptible (DS-) TB is a 6-month regimen comprising isoniazid, rifampicin, pyrazinamide, and ethambutol (HRZE) or a 4-month regimen comprising isoniazid, rifapentine, pyrazinamide, and moxifloxacin (HPZM). Significant challenges exist with regard to shortening the duration of treatment and improving treatment outcomes for DS-TB, as well as isoniazid-monoresistant (HR-) TB. New oral 6-month regimens combining bedaquiline and pretomanid with linezolid, with or without moxifloxacin (BPaL and BPaLM), have recently become the standard of care for RR/MDR-TB, although their clinical utility is challenged by the toxicity of linezolid and the variable rate of fluoroquinolone resistance among RR/MDR-TB isolates. A 4-month regimen of BPaMZ was recently shown to prevent relapse in DS-TB patients but was associated with more hepatotoxicity than the HRZE control arm, which may be attributable to the co-administration of pyrazinamide and pretomanid (2). New drugs capable of replacing isoniazid in rifamycin-based regimens and contributing bactericidal and/or sterilizing activity to bedaquiline-based regimens (e.g., by replacing L, M, Pa, or Z) are needed to improve the treatment options for DS-, HR-, and RR/MDR-TB.

We hypothesized that an inhibitor of the *M. tuberculosis* β-ketoacyl synthase (KasA), an essential enzyme involved in mycolic acid synthesis, holds significant promise to impact the next generation of TB regimens. We previously reported the indazole sulfonamide JSF-3285 to be a potent inhibitor of KasA with significant efficacy at relatively low doses in acute and chronic mouse infection models of TB and additive activity with isoniazid or rifampicin (3). These findings, together with reports from GlaxoSmithKline (4), firmly established KasA as a high-value drug target. To further advance JSF-3285 as a preclinical candidate and KasA as a drug target, we performed additional *in vivo* efficacy studies in mouse models designed to evaluate the potential for JSF-3285 to contribute bactericidal and sterilizing activity toward improved regimens for DS-, HR-, and RR/MDR-TB.

## RESULTS

### Minimum inhibitory concentration (MIC) of JSF-3285 against *M. tuberculosis* H37Rv and isogenic isoniazid-resistant mutants

The mycolic acid synthesis inhibitors currently used in TB treatment, isoniazid and ethionamide/prothionamide, target the mycobacterial fatty acid synthase II (FAS-II) component enoyl-acyl carrier protein reductase InhA. By directly targeting KasA, which catalyzes the upstream reaction in the FAS-II pathway that affords the Claisen condensation product of an acyl-AcpM with a malonyl-AcpM unit (Acp = mycobacterial acyl carrier protein) (5), JSF-3285 should retain activity against *M. tuberculosis* isolates resistant to InhA inhibitors. The susceptibility of isoniazid-resistant clinical isolates to JSF-3285 was previously reported (3). However, the specific impact of *inhA* promoter mutations and *katG* mutations on susceptibility to JSF-3285 could not be determined from these diverse clinical isolates. To determine the specific impact of *inhA* promoter mutations and *katG* mutations conferring isoniazid resistance on susceptibility to JSF-3285, we determined the respective MICs of isoniazid and JSF-3285 against two previously characterized isogenic isoniazid-resistant mutants: an *inhA* promoter (−8T→A) mutant and a *katG* M1A mutant (6), alongside the parent H37Rv strain used in the following mouse efficacy experiments. The geometric mean MIC of JSF-3285 was similar to that of isoniazid against H37Rv but consistently lower than those of isoniazid against the isoniazid-resistant mutants (Table 1). As expected, JSF-3285 retained its activity against the *inhA* promoter and *katG* mutants, while the isoniazid MICs were ≥3 and ≥100 times higher, respectively, against these mutants compared to H37Rv. Although the specific mutations conferring resistance in the mutants tested here are not the most frequently encountered mutations in the clinical setting, these results support the potential utility of JSF-3285 as a component of novel regimens for the treatment of isoniazid-resistant strains.

**TABLE 1:** MICs of isoniazid and JSF-3285 against *M. tuberculosis* H37Rv and isogenic isoniazid-resistant mutants

	MIC (in μg/mL) against the indicated strain		
**Compound**	**H37Rv**	***inhA* promoter mutant**	***katG* mutant**
Isoniazid	0.076 [0.12, 0.06, 0.06]	0.25 [0.25, 0.25, 0.25]	>8
JSF-3285	0.095 [0.12, 0.06, 0.12]	0.12 [0.12, 0.12, 0.12]	0.12

^a^ Results against H37Rv and *inhA* promoter mutant are shown as geometric mean [MIC replicates].

### Experiment 1: Dose-ranging activity of JSF-3285 in a subacute mouse infection model of TB

**Fig 1:**
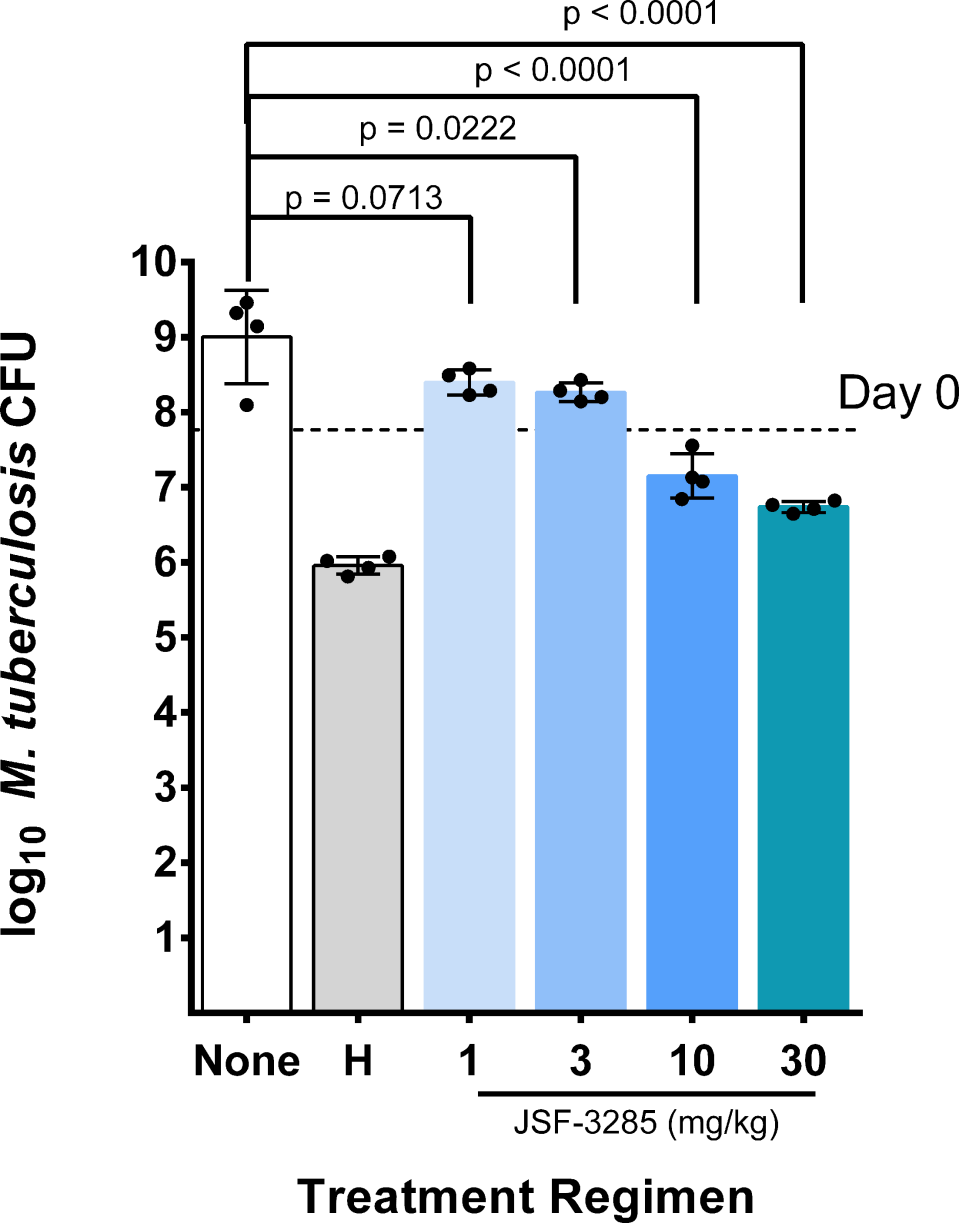
Mean (± SD) lung CFU counts after 1 month of treatment with increasing doses of JSF-3285 in the subacute mouse infection model. None, untreated (negative control). H, isoniazid 10 mg/kg (positive control). Points indicate CFU counts for individual mice. The dashed horizontal line indicates mean CFU count at Day 0.

The dose-ranging activity of JSF-3285 in acute and chronic mouse infection models of TB was previously reported (3). However, because we aimed to eventually investigate the contribution of JSF-3285 to novel drug regimens in long-term chemotherapy studies, including relapse after treatment as an endpoint, Experiment 1 was performed to evaluate the dose-ranging bactericidal activity of JSF-3285 monotherapy in a subacute high-dose aerosol infection model in BALB/c mice typically used for such studies to inform dose selection. The scheme of Experiment 1 is shown in Table S1. The aerosol infection implanted 4.45 ± 0.02 log_10_ colony-forming units (CFU) of *M. tuberculosis* in the lungs. At the start of treatment (14 days post-infection; D0), mice harbored a mean of 7.77 log_10_ CFU in the lungs. Whereas untreated mice (negative control) reached a humane endpoint requiring euthanasia at 24 days post-infection, all mice treated with isoniazid (H) (10 mg/kg) (positive control) or JSF-3285 (1, 3, 10, or 30 mg/kg), administered once daily (QD), 5 days per week, survived to the predetermined efficacy endpoint at 6 weeks post-infection (i.e, after 4 weeks of treatment). Mice receiving JSF-3285 at 1 mg/kg had a lower (*P* = 0.0713) mean lung CFU count compared to the euthanized untreated mice but a significantly higher (*P* = 0.0025) mean CFU count (a 0.68 log_10_ increase) when compared with mice sacrificed at D0 (Fig. 1). Mice treated with the 3 mg/kg dose of JSF-3285 had significantly lower CFU counts when compared with the untreated mice (*P* = 0.0222) but not (*P* = 0.1511) when compared to Day 0 mice (0.50 log_10_ decrease vs Day 0), indicating a bacteriostatic effect. In mice receiving the higher doses of JSF-3285, 10 and 30 mg/kg, significant reductions in mean CFU were observed when compared with those of untreated mice (*P* = 0.0188 and *P* < 0.0001, respectively); and when compared with D0 mice (0.62 and 1.02 log_10_ decreases; *P* = 0.0040 and *P* < 0.0001, respectively), indicating a bactericidal effect. However, all JSF-3285 doses were significantly less active than those of isoniazid, which reduced the mean CFU count by 1.80 log_10_ compared to Day 0 (*P* < 0.0001).

### Experiment 2a: Impact of dose fractionation of JSF-3285 monotherapy

To determine the effects of different dosing schedules on the activity of JSF-3285 and better understand PK-PD relationships for optimizing nonclinical and clinical dosing regimens, a dose fractionation study was performed in an acute mouse infection model. This model was selected for its large dynamic range, which makes it well suited to discriminate differences between dosing groups. The scheme for Experiment 2 is shown in Table S2. Mice were aerosol-infected with 3.84 ± 0.03 log_10_ CFU of *M. tuberculosis* H37Rv, and the treatment was started 6 days post-infection. Four different total weekly doses: 18, 60, 180, and 600 mg/kg per week, were each divided into twice daily (BID; 6 hours apart), QD, or thrice weekly (QOD) dosing schedules. An additional arm was intended to receive a total weekly dose of 2,400 mg/kg administered as 200 mg/kg BID. A positive control arm received isoniazid 10 mg/kg QD. Untreated mice served as negative controls. Dosing occurred 6 days per week (Monday through Saturday) for 3 weeks, except that isoniazid was administered 5 days/week. Mice receiving JSF-3285 at 200 mg/kg BID developed ruffled fur and reduced activity during the first week of dosing and were switched to 200 mg/kg QD for the last 2 weeks of treatment.

Infected mice were sparsely sampled during the last week of treatment for small-volume blood samples by submandibular puncture at 2 and 24 hours post-dose. Results were consistent with those of previously reported exposures in uninfected mice (3). At 2 hours post-dose, mean JSF-3285 concentrations exceeded the MIC (95 ng/mL) by at least an order of magnitude in all nine QD and BID dosing arms (Fig. S1). At 24 hours post-dose, only doses ≥ 100 mg/kg/day maintained trough concentrations above the MIC.

**Fig 2:**
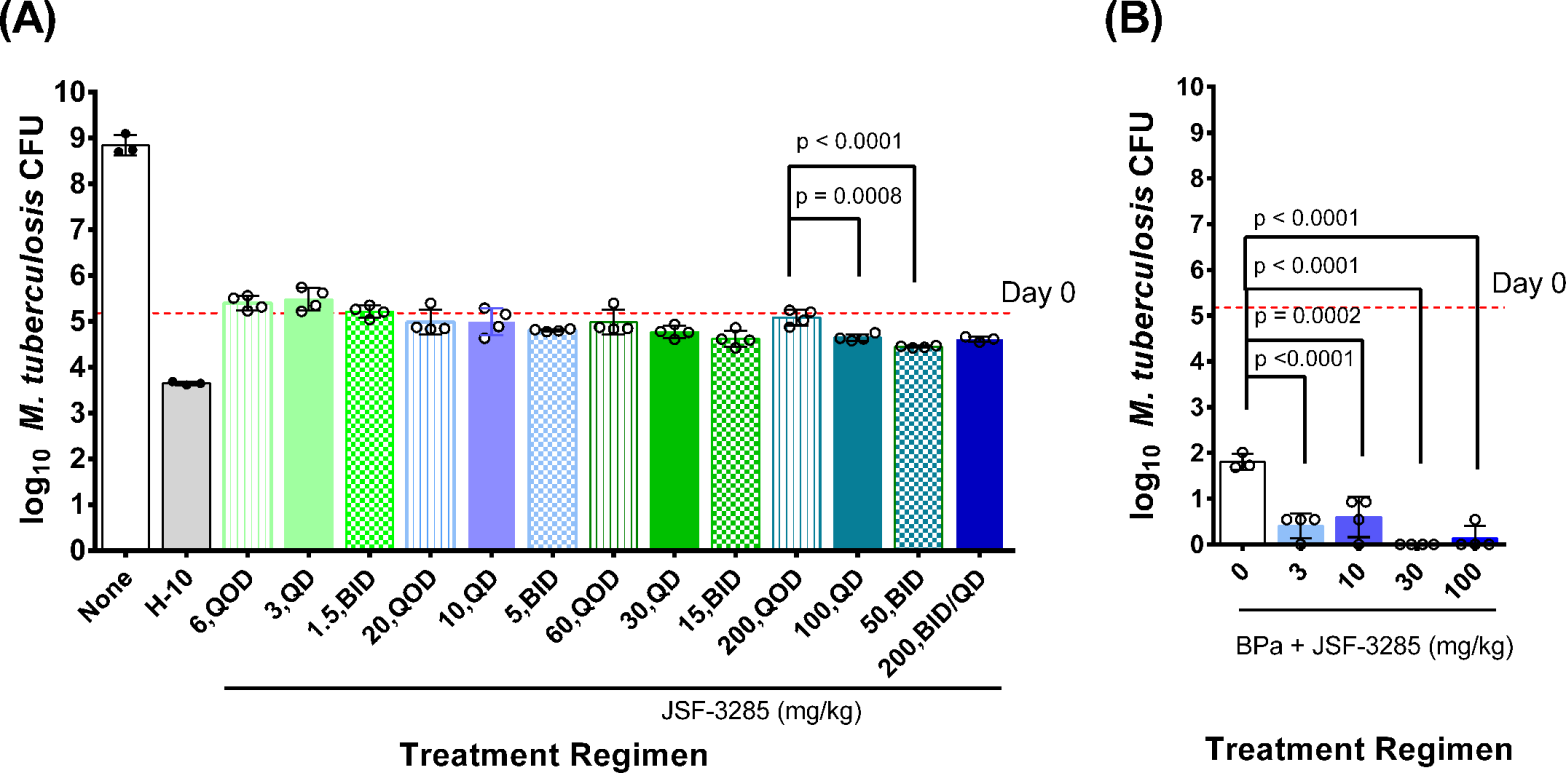
Mean (± SD) lung CFU counts after 3 weeks of treatment in Experiment 2a evaluating the dose fractionation of JSF-3285 in monotherapy (**A**); and in Experiment 2b evaluating the dose-ranging activity of JSF-3285 in combination with bedaquiline (25 mg/kg) and pretomanid (50 mg/kg) (BPa) (**B**). None, untreated (negative control). H, isoniazid 10 mg/kg (positive control). Points indicate CFU counts for individual mice. Dashed horizontal lines indicate mean CFU count at Day 0. The lower limit of detection was 2.5 CFU, which translates to 0.54 log10 after adding 1 prior to log transformation. The treatment regimen indicated as 200 BID/QD was 200 mg/kg BID for 1 week and then 200 mg/kg QD for 2 weeks.

The mean lung CFU count in untreated mice increased from 5.18 log_10_ at D0 to 8.84 log_10_ at D18, when untreated mice reached a humane endpoint and required euthanasia. Treated mice survived to the predetermined efficacy endpoint at Day 21. As shown in Fig. 2A, isoniazid treatment reduced the mean CFU count by approximately 1.5 log_10_ from D0. The net effect of JSF-3285 monotherapy was largely bacteriostatic over the range of doses tested in this acute infection model, with even the lowest weekly dose level preventing any significant increase in CFU counts compared to D0 and the highest weekly dose levels reducing the mean CFU count by approximately 0.5 log_10_ CFU. At each total weekly dose level of 18, 60, and 180 mg/kg, no significant difference in activity was observed irrespective of whether JSF-3285 was administered BID, QD, or QOD, although a trend toward greater activity with more frequent dosing was observed. At a total weekly dose level of 600 mg/kg, QD dosing and BID dosing were significantly more effective than QOD dosing (*P* = 0.0008 and *P* < 0.0001, respectively). Based on the general dose proportionality in exposure previously observed at daily doses ranging from 5 to 300 mg/kg (3) and assuming that the three dosing schedules would therefore result in a similar weekly area under the concentration–time curve (AUC) values, these results suggest that the efficacy correlates with AUC and may also be enhanced by optimizing the time above the MIC.

**Fig 3:**
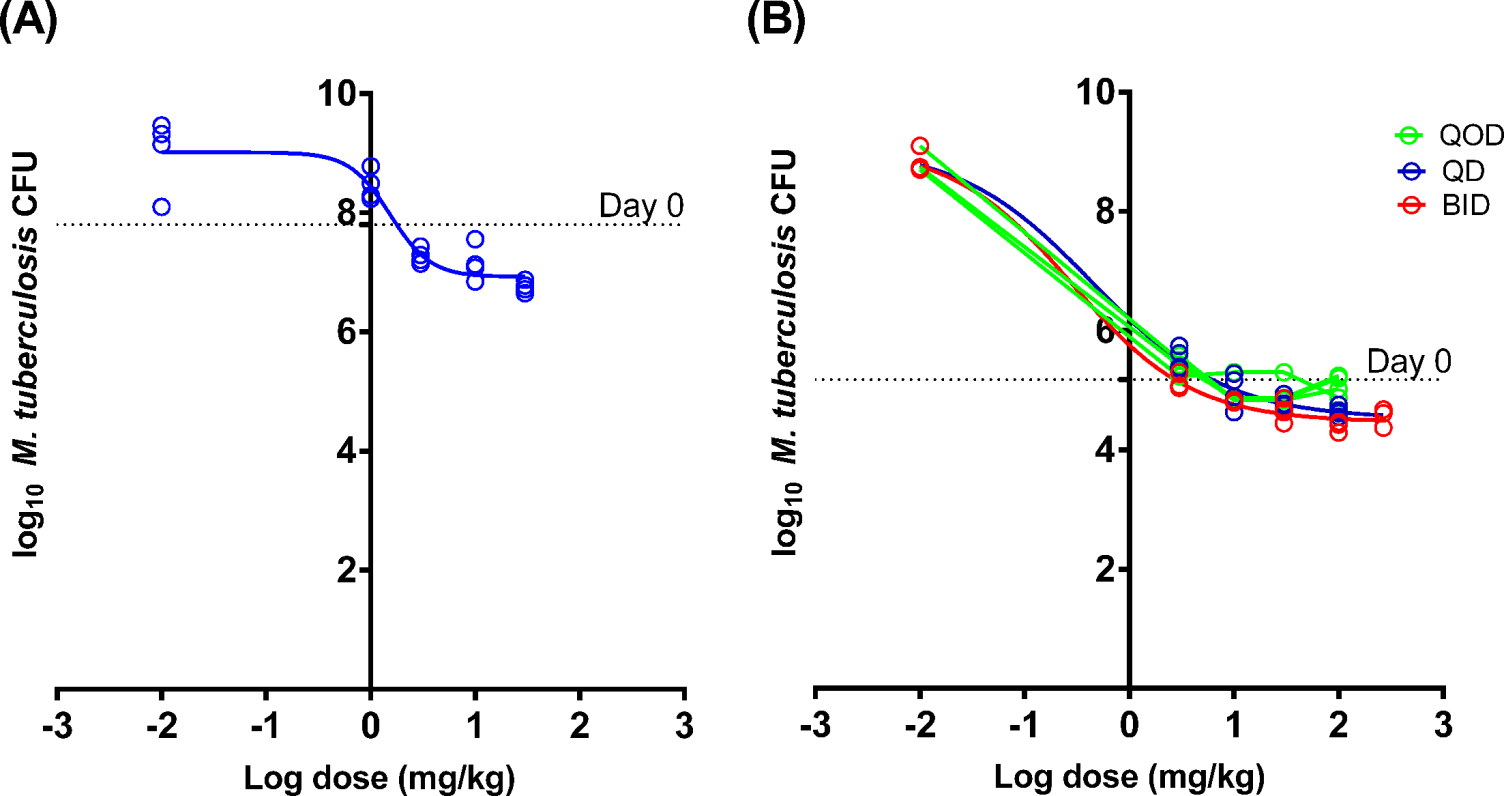
Dose–response relationships for JSF-3285 monotherapy in the subacute infection model in Experiment 1 (**A**) and the acute infection model in Experiment 2a (**B**). Dashed horizontal lines indicate the mean CFU count at Day 0. A dose of 0.01 mg/kg was substituted for 0 in untreated controls to enable log transformation and curve fitting.

From Experiment 1 (dose-ranging in the subacute infection mouse model) and Experiment 2a (acute mouse infection model), the dose–response relationships for JSF-3285 were derived and are shown in Fig. 3. Table 2 shows the estimated ED_50_ and ED_90_ values. The results of the two experiments were consistent in suggesting an ED_90_ between 4 and 11 mg/kg/day.

**TABLE 2:** Estimated ED_50_ and ED_90_ parameters from Experiments 1 and 2a

Experiment, dosing frequency	Model	ED_50_ (mg/kg)	ED_90_ (mg/kg)
Experiment 1, QD	Subacute (14-day)	1.5	4.2
Experiment 2a, QD	Acute (6-day)	0.4	11.3
Experiment 2a, BID	Acute (6-day)	0.3	5.2

#### Experiment 2b: Dose-ranging activity of JSF-3285 in combination with bedaquiline and pretomanid

The utility of the BPaL regimen is limited by the poor tolerability of linezolid. We previously found that the addition of isoniazid or a direct-acting InhA inhibitor significantly increased the activity of the BPa combination, suggesting that a KasA inhibitor could have a similar effect. In parallel with the dose fractionation study (Experiment 2a), we explored the dose-ranging activity of JSF-3285 when combined with BPa. The scheme of the experiment is shown in Table S2. Beginning on D0, mice received BPa plus either JSF-3285 at one of four doses (3, 10, 30, or 100 mg/kg QD) or the JSF-3285 vehicle administered 4 hours after BPa. Dosing was 5 days per week for 3 weeks. As shown in Fig. 2B, regimens adding JSF-3285 at any dose (3, 10, 30, or 100 mg/kg) to BPa were significantly more active than BPa alone (*P* ≤ 0.0002). Although the mean CFU counts in the various arms receiving BPa +JSF-3285 were not significantly different from each other, 30 mg/kg was the lowest JSF-3285 dose to render all or most mice culture-negative.

#### Experiment 3: Bactericidal activity of JSF-3285 in combination with existing first- and second-line TB drugs

To further assess the potential contribution of JSF-3285 to drug combinations with proven sterilizing activity in the subacute mouse infection model more commonly used for combination studies, we tested the effect of adding JSF-3285 to each of the following two-drug backbones: (1) rifapentine plus pyrazinamide (PZ) (2), BPa, and (3) bedaquiline plus pyrazinamide (BZ). To identify promising four-drug JSF-3285-containing combinations, we also tested the effect of adding a fourth drug (e.g., H, Z, moxifloxacin [M], clofazimine [C], or linezolid [L]) to these three-drug JSF-3285-containing combinations. PZMH, BPaL, and BPaMZ were included as clinical comparators (2, 7–9). The scheme for this experiment is shown in Table S3. JSF-3285 was dosed at 30 mg/kg once daily as this dose was approximately three times the estimated ED_90_ and the lowest dose to render the majority of mice culture-negative when combined with BPa in Experiment 2b. All three backbones (PZ, BPa, and BZ) tested with one or two additional drugs were assessed after 1 month of treatment, while only the two less active backbones (PZ and BPa) tested with one or two additional drugs were assessed after 1.5 months of treatment.

**Fig 4:**
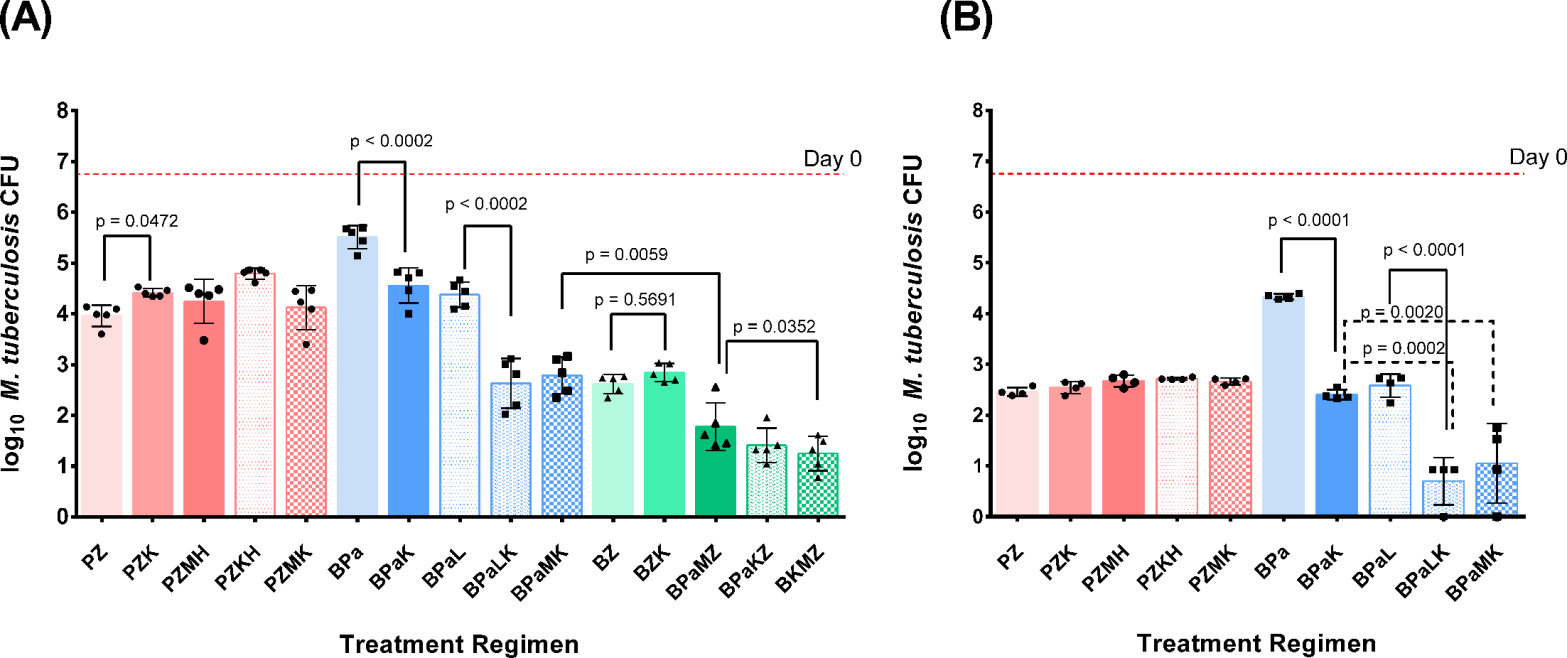
Mean (± SD) lung CFU counts after 1 month (**A**) and 1.5 months (**B**) of treatment in Experiment 3. Red bars show data from groups receiving regimens containing PZ, blue bars show data from groups receiving regimens containing BPa but not Z, and green bars show data from groups receiving regimens containing BZ. K = JSF-3285 30 mg/kg, P = rifapentine 10 mg/kg, Z = pyrazinamide 150 mg/kg, H = isoniazid 10 mg/kg, M = moxifloxacin 100 mg/kg, B = bedaquiline 25 mg/kg, Pa = pretomanid 50 mg/kg, and L = linezolid 50 mg/kg. Points indicate CFU counts for individual mice. Dashed horizontal lines indicate mean CFU count at Day 0. The lower limit of detection was 2.5 CFU, which translates to 0.54 log10 after adding 1 prior to log transformation.

After 4 weeks of treatment, JSF-3285 did not exhibit additive activity when combined with PZ or BZ. In fact, the addition of JSF-3285 modestly antagonized the activity of PZ after 1 month of treatment (*P* = 0.0472) (Fig. 4A) but not after 6 weeks of treatment (Fig. 4B). The combinations of PZ + JSF-3285 with either moxifloxacin or clofazimine were just as effective as the clinical comparator PZMH at both time points. However, when both isoniazid and JSF-3285 were combined with PZ, an additive antagonistic effect was noted, such that PZMH was significantly more active than the combination of PZ + JSF-3285 with isoniazid (*P* = 0.0058) after 1 month, but not after 6 weeks. As observed with the PZ backbone, the addition of JSF-3285 did not increase the activity of BZ (*P* = 0.5691). However, compared to the four-drug clinical comparator BPaMZ, the four-drug combination replacing Pa with JSF-3285 (i.e., BKMZ) was more active (*P* = 0.0352), while the combination replacing M with JSF-3285 (i.e., BPaKZ) was not statistically significantly superior. Replacing Z with JSF-3285 (i.e., BPaMK) was less effective than BPaMZ (*P* = 0.0059). In contrast to its lack of additivity with the PZ and BZ backbones, the addition of JSF-3285 significantly increased the activity of BPa and BPaL (*P* ≤ 0.0002 for each comparison at both time points). BPa +JSF-3285 was as effective as the clinical comparator BPaL, while the regimen combining BPa +JSF-3285 with moxifloxacin was superior to that of BPaL (*P* < 0.001 at both time points).

#### Experiment 4: Contribution of JSF-3285 to the sterilizing activity of novel combinations in a mouse relapse model

Following confirmation of the additive effects of JSF-3285 to BPa and BPaL (in Experiments 2 and 3) and the strong bactericidal activity of JSF-3285 in combination with BPaM and BMZ (in Experiment 3), we carried out Experiment 4 to confirm that adding JSF-3285 increases the sterilizing activity of each of these regimen backbones in a relapse mouse model. Mice were treated for 1.5, 2, or 2.5 months (6, 8, or 10 weeks) and then held for 3 months without treatment before being sacrificed to assess for relapse (10). The scheme for the experiment is shown in Table S4.

Unlike the statistically significant CFU reductions observed when JSF-3285 was added to BPa and BPaL in Experiment 3, the CFU reductions observed with the same regimen modification did not reach statistical significance after 1 month of treatment in Experiment 4 (Table 3). However, BPaMK was significantly more active than BPaM alone or BPaK (*P* < 0.0001), indicating that both JSF-3285 and M contribute to the activity of the BPaMK regimen. BMZK was not significantly more active than BMZ. However, BPaMK and BMZK (where JSF-3285 replaces Z and Pa, respectively, in BPaMZ) were not significantly different from BPaMZ.

**TABLE 3:** Efficacy of regimens adding JSF-3285 to selected 2- or 3-drug combinations in Experiment 4^*a*^

Regimen	Mean log_10_ CFU count (±SD)			Proportion of mice showing relapse 3 months after treatment for		
	W-2	D0	Month 1	1.5 months	2 months	2.5 months
Untreated	4.15 ± 0.03	7.05 ± 0.01				
BPa			4.88 ± 0.28	NT	NT	15/15
BPaL			3.69 ± 0.44	NT	12/15	9/15*c*
BPaK			4.44 ± 0.32	NT	14/15	8/15^*d*^
BPaLK			3.27 ± 0.43	NT	4/15^4^	1/15^*e*^
BPaM			4.61 ± 0.12	NT	NT	NT
BPaMK			2.47 ± 0.34^*b*^	NT	4/15	0/15
BPaMZ			2.23 ± 0.44	11/15	NT	NT
BMZ			2.42 ± 0.30	12/15	1/15	NT
BMZK			1.99 ± 0.36	13/15	1/15	NT

^*a*^ NT, not tested.

^*b*^ *P*<0.0001 vs BPaM.

^*c*^ *P*<0.05 vs BPa.

^*d*^ *P*<0.01 vs BPa.

^*e*^ *P*<0.01 vs BPaL.

The addition of JSF-3285 to BPa significantly reduced the proportion of mice showing relapse after 2.5 months of treatment compared to treatment with BPa, and BPaK was just as effective as BPaL in terms of sterilizing activity. Likewise, the addition of JSF-3285 to BPaL significantly reduced the proportion of mice showing relapse after 2 and 2.5 months of treatment compared to treatment with BPaL. BPaMK was also superior to BPaL and had a sterilizing activity nearly identical to that of BPaLK. While BPaMK resulted in more relapses after 2 months of treatment compared to BPaMZ, the difference was not statistically significant. The addition of JSF-3285 to BMZ did not increase the sterilizing activity of the combination, but neither did the addition of pretomanid. Therefore, BMZK was as bactericidal and sterilizing as BPaMZ (*P* = 0.9999).

#### Experiment 5: Contribution of JSF-3285 to the bactericidal activity of additional combinations of first- and second-line TB drugs

Experiment 5 tested the effects of addition of JSF-3285 to other novel three-drug combinations based on bedaquiline plus two other drugs currently used for TB treatment, including pretomanid, rifabutin (Rb), clofazimine, moxifloxacin, and pyrazinamide. The investigational new DprE1 inhibitor TBA-7371 (A), which recently completed a Phase 2 clinical trial (*ClinicalTrials.gov* Identifier: NCT04176250), was also included. Specifically, we evaluated the contribution of JSF-3285 to the following combinations: BPaRb, BRbA, BCM, BCA, BCRb, BMRb, BZRb, and BCZ. BPaL and BPaMZ were included as clinical comparators. The regimen efficacy was assessed by lung CFU counts after 1 and 2 months of treatment, except that BCRb and the Z-containing regimens were only assessed after 1 month due to the strong bactericidal activity of the backbone regimens. The scheme for Experiment 5 is given in Table S5.

**Fig 5:**
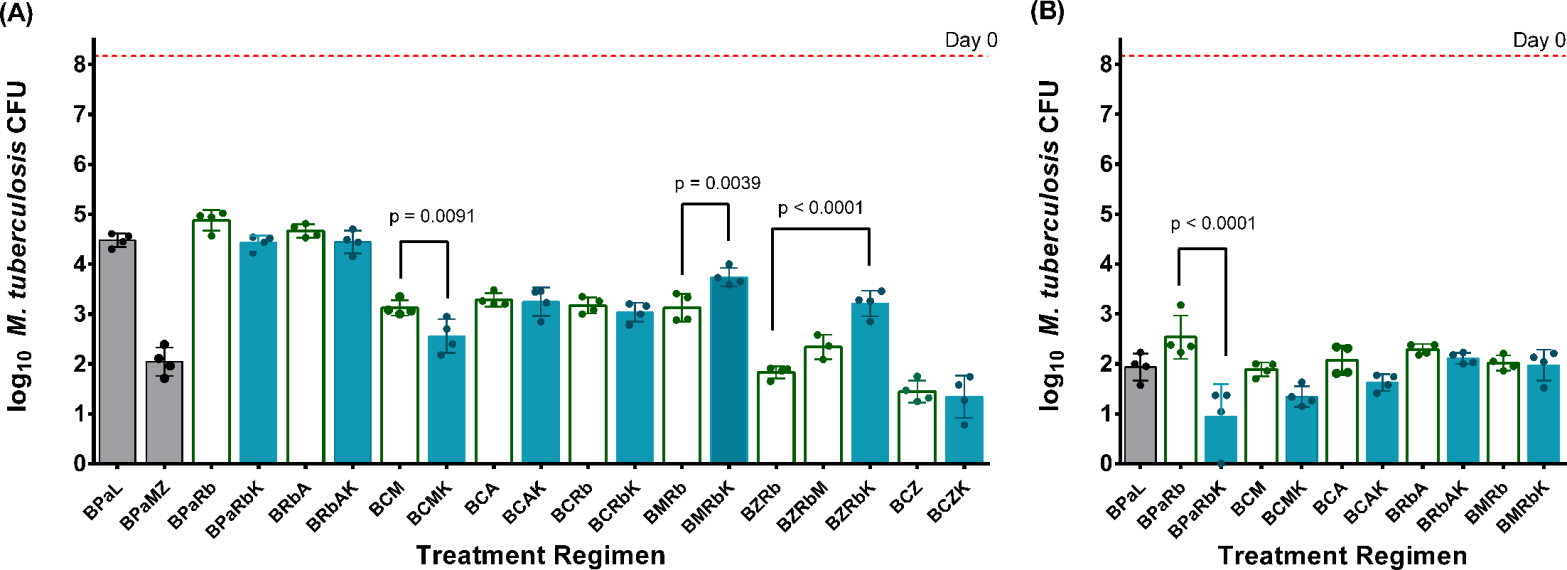
Mean (± SD) lung CFU after 1 month (**A**) and 2 months (**B**) of treatment in Experiment 5. Gray bars show data from the control regimens, BPaL and BPaMZ, open bars show data from test regimen backbones, and blue bars show data from groups receiving test regimen backbones plus JSF-3285. K = JSF-3285 30 mg/kg, *P* = rifapentine 10 mg/kg, Z = pyrazinamide 150 mg/kg, H = isoniazid 10 mg/kg, M = moxifloxacin 100 mg/kg, B = bedaquiline 25 mg/kg, Pa = pretomanid 50 mg/kg, L = linezolid (50 mg/kg), Rb = rifabutin (5 mg/kg), C = clofazimine (6.25 mg/kg), and A = TBA-7371 (200 mg/kg). Points indicate CFU counts for individual mice. Dashed horizontal lines indicate mean CFU count at Day 0. The lower limit of detection was 2.5 CFU, which translates to 0.54 log10 after adding 1 prior to log transformation.

As shown in Fig. 5A, after 1 month of treatment, BCM +JSF-3285 was significantly more active than BCM (*P* = 0.0091), whereas the addition of JSF-3285 to BRbA, BCZ, BCA, and BCRb did not significantly alter the activity of these regimens (*P* > 0.9999). On the other hand, the addition of JSF-3285 significantly reduced the activities of BMRb (*P* = 0.0039) and BZRb (*P* < 0.0001). After 2 months, the additive effect of JSF-3285 with BCM was maintained but did not quite reach statistical significance (*P* = 0.0857) (Fig. 5B). Although BPaRb +JSF-3285 was not significantly more active than BPaRb (*P* = 0.1599) after the first month of treatment, it was significantly more active than BPaRb (*P* < 0.0001) after 2 months of treatment, where one mouse receiving BPaRb +JSF-3285 had a CFU count below the limit of detection of 2.5 CFU. It was also notable that the addition of JSF-3285 to BMRb was no longer antagonistic after 2 months of treatment. All JSF-3285-containing combinations, except those with BPaRb and BRbA, were significantly more active than BPaL (*P* < 0.0001) after 1 month of treatment, whereas only BPaRb +JSF-3285 was significantly more active than BPaL (*P* = 0.0028) after 2 months of treatment. Compared to BPaMZ, all of the combinations without Z (except for BCM +JSF-3285; *P* = 0.0717) were significantly less active at M1 (*P* > 0.0001). BZRb and BZRbM were statistically not different from BPaMZ (*P* > 0.9999), while BCZ and BCZ +JSF-3285 were significantly more active than BPaMZ (*P* = 0.0226 and *P* = 0.0046, respectively).

## DISCUSSION

The most significant finding reported here is that the addition of the KasA inhibitor JSF-3285 increases the bactericidal activity of the BPa and BPaL regimen backbones and shortens the treatment duration needed to prevent relapse in a well-established mouse model of TB. The 6-month BPaL regimen achieved high rates of successful treatment outcomes in the Nix-TB, ZeNix, and TB-PRACTECAL trials (8, 9, 11). In TB-PRACTECAL, the addition of moxifloxacin to BPaL resulted in faster sputum culture conversion and may have reduced the risk of relapse with acquired bedaquiline resistance (12). BPaL(M) is now recommended by the WHO for treatment of RR/MDR-TB, but its utility is limited by linezolid toxicity and by resistance to moxifloxacin in some RR/MDR-TB isolates. The finding that BPa +JSF-3285 has sterilizing efficacy comparable to that of BPaL in this study suggests that combining a KasA inhibitor such as JSF-3285 with BPa could provide an effective alternative regimen or regimen backbone to BPaL for RR/MDR-TB and, if sufficient safety is demonstrated, DS-TB. We recently reported similar additive effects on the bactericidal and sterilizing activity of BPa-based combinations for a direct-acting InhA inhibitor (13), providing further support for inhibition of FAS-II as a means for improving the efficacy of regimens based on a diarylquinoline-nitroimidazole backbone.

Importantly, the addition of moxifloxacin to BPa +JSF-3285 further increases the efficacy, yielding a four-drug regimen with efficacy approaching that of BPaMZ. Likewise, BMZ + JSF-3285 had a sterilizing efficacy comparable to that of BPaMZ. By replacing pyrazinamide or pretomanid with JSF-3285, BPaM+JSF-3285 or BMZ+JSF-3285 could provide alternatives to BPaMZ, which was clinically efficacious as a 4-month regimen in the SimpliciTB trial but with a rate of hepatotoxicity that precludes its routine use (2).

BPaL(M) is now recommended as the standard of care for RR/MDR-TB. Thus, it is important to compare BPaL(M) with BPa+JSF-3285, BPaL+JSF-3285, and BPaM+JSF-3285 in a C3HeB/FeJ mouse model or another animal model that develops caseating lung lesions different from the cellular lung lesions found in the BALB/c mouse models evaluated here. Such an experimental design would further confirm the potential of JSF-3285 to replace moxifloxacin and linezolid in the BPaL(M) regimen.

The dose-ranging and dose fractionation studies performed here confirmed the potent anti-tubercular activity of JSF-3285, establishing ED_90_ values of approximately 5–10 mg/kg/day. Although the maximal effects of JSF-3285 were not as great as those of isoniazid as monotherapies in the acute and subacute mouse infection models studied here, which could relate to the somewhat slower time-kill kinetics of JSF-3285 previously observed *in vitro* (3), JSF-3285 has shown bactericidal effects of a similar magnitude to isoniazid in chronic mouse infection models, in which the proportion of actively replicating bacteria is lower (3). The significant contribution of JSF-3285 to the BPa backbone at doses as low as 3 mg/kg in the acute infection model in Experiment 2b is likely due in part to the rapid bacteriostatic effects of JSF-3285 preventing bacterial multiplication, while the slower-onset bactericidal effects of the companion drugs and the mouse’s own adaptive immune response are mounting. Further delineation of the exposure–response relationships governing the sterilizing activity of JSF-3285 in combination with BPa is warranted to better inform clinical dose predictions.

Despite the clear contribution of JSF-3285 to the bactericidal and sterilizing activity of regimens containing BPa, BPaL, and BPaM, the addition of JSF-3285 to other drug combinations had no significant effect or was even antagonistic. Most notably, the addition of JSF-3285 to PZ significantly increased the mean lung CFU count after 4, but not 6, weeks of treatment; and the addition of isoniazid to PZ +JSF-3285 further increased the week 4 CFU counts. While the basis for the antagonism of PZ by JSF-3285 and isoniazid is not known, similar antagonistic effects are reproducibly observed with the addition of isoniazid to rifampin-pyrazinamide-containing regimens in our model (6, 14), suggesting that the antagonistic effects of the two FAS-II inhibitors may be mechanistically related. The antagonistic effect of JSF-3285 on another rifamycin-pyrazinamide combination, BZRb, may be similarly related. However, since the addition of JSF-3285 to BMRb also significantly increased the mean lung CFU count after 4, but not 6, weeks of treatment, a drug–drug interaction affecting the exposures of Rb or JSF-3285 or both could be considered another explanation.

## MATERIALS AND METHODS

### Bacterial strains

*M. tuberculosis* H37Rv was mouse-passaged, frozen in aliquots, and sub-cultured in Middlebrook 7H9 broth supplemented with 10% oleic acid-albumin-dextrose-catalase (OADC) (Fisher, Pittsburgh, PA) and 0.05% Tween 80 prior to aerosol infection. Isogenic isoniazid-resistant *M. tuberculosis* mutants harboring either an *inhA* promoter (−8T→A) mutation or a KatG M1A mutation were selected in mice infected with *M. tuberculosis* H37Rv and treated with isoniazid, as previously described (6). These isolates were propagated at 37^°^C in Middlebrook 7H9 broth (Difco) supplemented with 10% OADC, 0.2% glycerol, and 0.5% Tween 80.

### Drugs and experimental compounds

The tested drugs were sourced from commercial suppliers. JSF-3285 was synthesized utilizing a previously reported route (3).

### Determination of the MIC against *M. tuberculosis*

The broth macrodilution method in complete 7H9 media without Tween 80 was used to determine the MICs of isoniazid and JSF-3285 against *M. tuberculosis* H37Rv and isogenic isoniazid-resistant mutants harboring either an *inhA* promoter (−8T→A) mutation or a KatG M1A mutation. The assays were performed using doubling dilutions and the following concentration ranges: 0.015–1 µg/mL against H37Rv, 0.0625–4 µg/mL against the *inhA* promoter mutant, and 0.0625–8 µg/mL against the KatG mutant. The MICs against the H37Rv parent strain and the *inhA* promoter mutant were measured thrice, with the KatG mutant included once.

### High-dose aerosol mouse infection model

All animal procedures were approved by the Animal Care and Use Committee of Johns Hopkins University. Six-week-old female BALB/c mice (Charles River Laboratories, Wilmington, MA) were infected with *M. tuberculosis* H37Rv using the inhalation exposure system (Glas-Col, Terre Haute, IN) and a fresh log-phase broth culture (optical density at 600 nm, 0.8 to 1.0), with the goal of implanting 4 log_10_ CFU in the lungs of each mouse. Treatment started 2 weeks later on D0 for Experiments 1, 3, 4, and 5. Treatment started (D0) 1 week after infection in Experiment 2. Four or five mice were humanely killed 1 day after infection and on D0 to determine the number of bacteria implanted in the lungs and the number at the start of treatment, respectively.

### Drug treatment

Mice were treated with a single drug or combinations of drugs at the following doses (in mg/kg body weight) administered by gavage: bedaquiline (25), clofazimine (6.25), isoniazid (10), linezolid (100), moxifloxacin (100), pretomanid (50), pyrazinamide (150), rifabutin (5), rifapentine (10), and TBA-7371 (100). All dosing was once daily, 5 days per week (Mon–Fri), except as noted for Experiment 2a below. For the dose-ranging monotherapy study (Experiment 1), JSF-3285 was administered at doses of 1, 3, 10, and 30 mg/kg. For the monotherapy dose fractionation study (Experiment 2a), JSF-3285 was administered at total weekly doses ranging from 18 to 2,400 mg/kg for 3 weeks, divided into twice daily (BID), once daily (QD), or thrice weekly (Mon–Wed–Fri; QOD) dosing schedules and administered Mon–Sat. For the dose-ranging study in combination with BPa in Experiment 2b, JSF-3285 was administered at doses of 3, 10, 30, and 100 mg/kg for 3 weeks. A JSF-3285 dose of 30 mg/kg was used when it was tested in all other multidrug therapy experiments (Experiments 3, 4, and 5). JSF-3285 was prepared in 0.5% carboxymethylcellulose and 0.5% wt/vol Tween 80. The other drugs were formulated as previously described (10, 15–17): isoniazid, moxifloxacin, and pyrazinamide in distilled water, bedaquiline in an acidified 10% (2-hydroxypropyl)-b-cyclodextrin solution, pretomanid in a 20% HPCD-lecithin micelle (CM-2) formulation; clofazimine, rifabutin, and rifapentine in 0.05% (wt/vol) agarose solution, linezolid in 0.5% methylcellulose, and TBA-7371 in 0.5% carboxymethylcellulose with 0.5% (wt/vol) Tween 80. In combination studies, bedaquiline and pretomanid were administered in a single dose by mixing them together just before the gavage, and companion drugs were administered 4–6 hours afterward.

### Evaluation of the treatment efficacy

Evaluation of the treatment efficacy was based on lung CFU counts after 3, 4, 6, or 8 weeks of treatment (as specified in the experiment schemes); and, in Experiment 4, additional cohorts of mice were kept for 12 weeks after completing 6, 8, or 10 weeks of treatment to assess for relapse-free cure. At each time point, lungs were collected in 2.5 mL sterile PBS. Serial 10-fold dilutions of the lung homogenate were prepared and plated on 7H11 agar plates supplemented with 10% (vol/vol) OADC (Gibco), cycloheximide (10 mg/mL, Sigma), carbenicillin (50 mg/mL, Sigma), polymyxin B (25 mg/mL, Sigma), and trimethoprim (20 mg/mL, Sigma). Homogenates from mice receiving drug combinations were plated onto the same agar media, but with the addition of activated charcoal powder (0.4%, wt/vol) to reduce drug carryover and doubling the concentration of selective antibiotics in the media to mitigate binding to charcoal. Colonies were counted after 4 and 6 weeks of incubation at 37°C to ensure that all cultivable bacteria would be detected.

### Plasma pharmacokinetics analysis

Whole blood was collected into BD Microtainer tubes containing lithium heparin via submandibular puncture. Plasma was separated by centrifugation at 10,000 rpm for 5 minutes and frozen at −80°C before analysis. JSF-3285 was quantified by liquid chromatography-tandem mass spectrometry (LC-MS/MS), as previously described (3).

### Statistical analysis

Lung CFU counts (x) were log-transformed (as x + 1) before analysis. Group mean CFU counts were compared using one-way ANOVA with Dunnett’s or Bonferroni’s multiple comparison tests, as appropriate. The proportions of relapse in mice were compared using Fisher’s exact test. Sigmoidal (4-variable) inhibitory dose–response curves were fitted by nonlinear regression modeling. Because 0 cannot be log-transformed, a dose value of 0.01 mg/kg was substituted for 0 to enable curve fitting. The sensitivity of the ED50 and ED90 estimates to this assumption was explored by substituting a range of values from 0.1 to 0.001 mg/kg for 0 in Experiment 2. The ranges of ED_50_ and ED_90_ estimates for QD dosing were 0.4–0.9 and 6–12 mg/kg, respectively, and the ranges for BID dosing were 0.2–0.7 and 4–7 mg/kg, respectively. All analyses were performed with GraphPad Prism, version 5 (GraphPad, San Diego, CA).

## SUPPLEMENTAL MATERIAL

Fig. S1 aac.00130-25-s0001.tif Mean ({plus minus} SD) plasma concentrations of JSF-3285 during QD dosing (A) and BID dosing (B).

Supplemental material aac.00130-25-s0002.docx Tables S1 to S5; Supplemental figure caption.

ASM does not own the copyrights to Supplemental Material that may be linked to, or accessed through, an article. The authors have granted ASM a non-exclusive, world-wide license to publish the Supplemental Material files. Please contact the corresponding author directly for reuse.
