# Supplementary material for: The KasA inhibitor JSF-3285 improves the sterilizing activity of bedaquiline-pretomanid-containing regimens in a mouse model of tuberculosis
Source: Antimicrob Agents Chemother. 2025 Apr 23;69(6):e00130-25. doi: 10.1128/aac.00130-25 (PMC12135538; doi:10.1128/aac.00130-25)
Supplement: Supplemental material — Tables S1 to S5; Supplemental figure caption. [file aac.00130-25-s0002.docx]

Table S1: Scheme for Experiment 1.

|  | Timepoint and number of mice for CFU | | | |
| --- | --- | --- | --- | --- |
| **Regimen*** | **D-13** | **D0** |  | **M1** |
| Untreated | 2 | 3 |  | 4 |
| INH_10_ |  |  |  | 4 |
| JSF-3285_1_ |  |  |  | 4 |
| JSF-3285_3_ |  |  |  | 4 |
| JSF-3285_10_ |  |  |  | 4 |
| JSF-3285_30_ |  |  |  | 4 |

*The numbers in subscript indicate dose in mg/kg body weight.

Table S2: Scheme for Experiments 2a and 2b.

| **Regimens** | | Timepoint and number of mice for CFU | | |
| --- | --- | --- | --- | --- |
| **Controls** |  | **D-6** | **D0** | **D21** |
| Neg. control | Untreated | 2 | 2 | 3 |
| Pos. control | H_10_ |  |  | 3 |
| ***Experiment 2a*** |  |  |  |  |
| **Total JSF-3285 dose over 6 days*** | JSF-3285 daily dose |  |  |  |
| 2400 mg/kg | 200_bid/qd_ |  |  | 4 |
| 600 mg/kg | 50_bid_ |  |  | 4 |
|  | 100_qd_ |  |  | 4 |
|  | 200_qod_ |  |  | 4 |
| 180 mg/kg | 15_bid_ |  |  | 4 |
|  | 30_qd_ |  |  | 4 |
|  | 60_qod_ |  |  | 4 |
| 60 mg/kg | 5_bid_ |  |  | 4 |
|  | 10_qd_ |  |  | 4 |
|  | 20_qod_ |  |  | 4 |
| 18 mg/kg | 1.5_bid_ |  |  | 4 |
|  | 3_qd_ |  |  | 4 |
|  | 6_qod_ |  |  | 4 |
| ***Experiment 2b*** |  |  |  |  |
| **BPa +**  **JSF-3285 dose ranging^**^** | 0 |  |  | 4 |
|  | 3 |  |  | 4 |
|  | 10 |  |  | 4 |
|  | 30 |  |  | 4 |
|  | 100 |  |  | 4 |

*^*^ Mice were dosed for 3 weeks, 6 days/week (Mon-Sat), for a total of 18 days of treatment, except that qod arms were dosed 3 days a week (M, W, F).*

***^**^*** *BPa+ JSF-3285 arms were dosed once daily for 3 weeks, 5 days/week (Mon-Fri), for a total of 15 days of treatment.*

Table S3: Scheme for Experiment 3

|  | Time point and number of mice for CFU | | | |  |
| --- | --- | --- | --- | --- | --- |
| **Treatment Regimen** | **D-13** | **D0** | **M1** | **M2** | **Total** |
| Untreated | 2 | 3 |  |  | 5 |
| PZHM |  |  | 5 | 4 | 9 |
| PZ |  |  | 5 | 4 | 9 |
| PZK |  |  | 5 | 4 | 9 |
| PZHK |  |  | 5 | 4 | 9 |
| PZKM |  |  | 5 | 4 | 9 |
| BPa |  |  | 5 | 4 | 9 |
| BPaL |  |  | 5 | 4 | 9 |
| BPaK |  |  | 5 | 4 | 9 |
| BPaLK |  |  | 5 | 4 | 9 |
| BPaMK |  |  | 5 | 4 | 9 |
| BPaMZ |  |  | 5 |  | 5 |
| BZ |  |  | 5 |  | 5 |
| BZK |  |  | 5 |  | 5 |
| BPaKZ |  |  | 5 |  | 5 |
|  | 2 | 3 | 70 | 40 | 115 |

P = rifapentine; Z = pyrazinamide, K = JSF-3285, H = isoniazid; B = bedaquiline; Pa = pretomanid; K = JSF-3285, L = linezolid; M = moxifloxacin.

Table S4: Scheme for Experiment 4

| **Regimen** | **Time point and number of mice for CFU endpoint** | | | **Time point and number of mice for relapse endpoint** | | |  |
| --- | --- | --- | --- | --- | --- | --- | --- |
|  | **D- 13** | **D0** | **M1** | **M1.5 (+3)** | **M2 (+3)** | **M2.5 (+3)** | **Total** |
| **Untreated** | 4 | 6 |  |  |  |  | **10** |
| **BPa** |  |  | 4 |  |  | 15 | **19** |
| **BPaL** |  |  | 4 |  | 15 | 15 | **34** |
| **BPaK** |  |  | 4 |  | 15 | 15 | **34** |
| **BPaLK** |  |  | 4 |  | 15 | 15 | **34** |
| **BPaM** |  |  | 4 |  |  |  | **4** |
| **BPaMK** |  |  | 4 |  | 15 | 15 | **34** |
| **BPaMZ** |  |  | 4 | 15 |  |  | **19** |
| **BMZ** |  |  | 4 | 15 | 15 |  | **34** |
| **BMZK** |  |  | 4 | 15 | 15 |  | **34** |
| **Total** | **4** | **6** | **32** | **45** | **90** | **75** | **256** |

Z = pyrazinamide, K = JSF-3285, B = bedaquiline; Pa = pretomanid; K = JSF-3285, L = linezolid; M = moxifloxacin.

Table S5: Scheme for Experiment 5

| **Regimen** | **Time point and number of mice for CFU** | | | |  |
| --- | --- | --- | --- | --- | --- |
|  | **D-13** | **D0** | **M1** | **M2** | **Total** |
| **BPaL** | 2 | 3 | 4 | 4 | 13 |
| **BPaMZ** |  |  | 4 |  | 4 |
| **BZRb** |  |  | 4 |  | 4 |
| **BZRbM** |  |  | 4 |  | 4 |
| **BZRbK** |  |  | 4 |  | 4 |
| **BZC** |  |  | 4 |  | 4 |
| **BZCK** |  |  | 4 |  | 4 |
| **BCRb** |  |  | 4 |  | 4 |
| **BCRbK** |  |  | 4 |  | 4 |
| **BPaRb** |  |  | 4 | 4 | 8 |
| **BPaRbK** |  |  | 4 | 4 | 8 |
| **BMRb** |  |  | 4 | 4 | 8 |
| **BMRbK** |  |  | 4 | 4 | 8 |
| **BCM** |  |  | 4 | 4 | 8 |
| **BCMK** |  |  | 4 | 4 | 8 |
| **BRbA** |  |  | 4 | 4 | 8 |
| **BRbAK** |  |  | 4 | 4 | 8 |
| **BCA** |  |  | 4 | 4 | 8 |
| **BCAK** |  |  | 4 | 4 | 8 |
| **Total** | 2 | 3 | 76 | 44 | 125 |

B = bedaquiline; Pa = pretomanid; L = linezolid; K = JSF-3285,

Rb = rifabutin; M = moxifloxacin; Z = pyrazinamide; C = clofazimine; A = TBA-7371.

Figure S1. Mean (± SD) plasma concentrations of JSF-3285 during QD dosing (A) and BID dosing (B). The group receiving 200 mg/kg BID for 1 week, then 200 mg/kg QD for 2 weeks (200 BID/QD) is shown in panel A. Dashed horizontal lines indicates the geometric mean MIC. The lower limit of quantification was 1 ng/ml.
